# Sustainable PA6 Composites from Recycled Carbon Fiber Thermoplastics for Mechanical and EMI Applications

**DOI:** 10.1021/acsomega.5c06965

**Published:** 2025-09-25

**Authors:** Ieda Cruz da Silva, Erick Gabriel Ribeiro dos Anjos, Larissa Stieven Montagna, Juliano Marini, Michelle Leali Costa, Mirabel Cerqueira Rezende, Jandro L. Abot, Fabio Roberto Passador

**Affiliations:** † Department of Science and Technology, 28105 Federal University of São Paulo (UNIFESP), 330 Talim St, São José dos Campos, São Paulo 12231-280, Brazil; ‡ Graduate Program in Materials Science and Engineering, 67828 Federal University of São Carlos (UFSCar), Rodovia Washington Luís,Km 235, São Carlos, São Paulo 13565-905, Brazil; § Department of Materials and Technology, São Paulo State University (UNESP), Av. Dr. Ariberto Pereira da Cunha 333, Guaratinguetá, São Paulo 12516-410, Brazil; ∥ Department of Mechanical Engineering, 8365 The Catholic University of America, District of Columbia, 620 Michigan Ave N.E, Washington 20064, United States

## Abstract

The growing demand for sustainable solutions in advanced composite materials has driven interest in reusing carbon fiber-reinforced thermoplastic (CFRT). This study investigates the rheological, mechanical, thermal, electrical, and electromagnetic properties of polyamide 6 (PA6) composites reinforced with recycled CFRT waste. The composites were prepared by the extrusion process with different ground CFRT contents (10 and 20 wt %). Differential scanning calorimetry (DSC) revealed that the presence of carbon fibers acts as an effective heterogeneous nucleating agent, increasing the crystallization temperature and the degree of crystallinity. Mechanical characterization demonstrated significant improvements, with the elastic modulus and ultimate tensile strength increasing by up to 80% and 69%, respectively, while strain at break was reduced due to the restriction of polymer chain mobility and stress concentration around fiber–matrix interfaces. Impact strength improved by 53% at higher ground CFRT contents, driven by energy dissipation mechanisms such as fiber pull-out and crack deflection. Electromagnetic characterization indicated promising shielding effectiveness (SET), reaching up to 16 dB in the X-band, with absorption-related mechanisms presented. The composites exhibit a desirable combination of mechanical performance and electromagnetic interference (EMI) shielding capabilities. These findings underscore the potential of CFRT-reinforced PA6 composites as sustainable, high-performance materials for applications requiring mechanical and electronic requirements.

## Graphical abstract

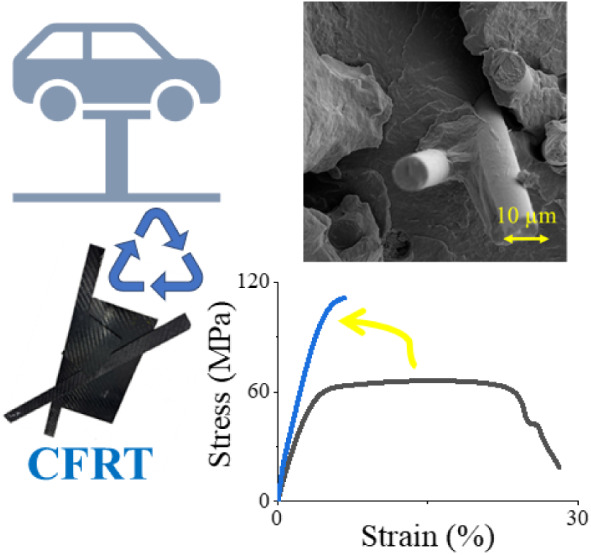

## 1 Introduction

The search for sustainable, environmentally friendly, and economically viable solutions for carbon fiber-reinforced thermoplastic (CFRT) waste has garnered scientific, academic, and industrial interest.^1^ In recent decades, the growing demand for CFRT has been driven by the aerospace, automotive, energy, oil and gas, defense, and sports goods industries, primarily due to their favorable combination of low weight and high mechanical strength, as well as their processing advantages, and notably, their recyclability.^2–4^

The choice of the thermoplastic matrix in a composite plays an important role in determining its thermal, mechanical, and chemical stability, durability, and suitability for advanced structural applications when combined with carbon fibers (CF).^5^ Among engineering thermoplastics, polyamide 6 (PA6) stands out as one of the most widely used matrices due to its good mechanical performance, cost-effectiveness, and broad applicability, particularly in the automotive and consumer goods industries. Additionally, PA6 can be processed at lower temperatures compared to other high-performance engineering thermoplastics, such as polyphenylene sulfide (PPS), polyetherketoneketone (PEKK), poly­(aryl ether ketone) (PAEK), polyetheretherketone (PEEK),^6,7^ and poly­(ether imide) (PEI).^8^

Thermoplastic composites offer the advantage of being recyclable, allowing them to remain within the production chain of the original sector or be redirected to secondary applications. However, in such second-generation uses, structural integrity is typically not retained, which limits their applicability in high-performance sectors.^9^ Thermal or chemical recycling processes, which enable the recovery of CF, are expensive and technically complex, requiring specialized equipment and trained personnel. Moreover, these methods pose environmental challenges: thermal recycling consumes large amounts of energy and emits pollutant gases, while chemical recycling relies on aggressive solvents, often rendering both approaches economically and environmentally challenging.^10,11^ Despite these challenges, several companies, such as ELG Carbon Fiber Ltd. (United Kingdom), Carbon Fiber Recycling Inc. (USA), and Vartega Recycled Carbon Fiber (USA), are already supplying good-quality recycled CF (rCF). Nonetheless, the rCF industry is still emerging, showing continuous progress and significant market potential.^12,13^

The mechanical recycling process enables the complete reuse of composite waste, both the matrix and reinforcement, through a physical procedure involving size reduction (grinding), followed by particle size separation.^14^ The resulting ground material can be reprocessed by extrusion, injection, or compression molding, often in combination with a thermoplastic matrix, to enhance the processability and mechanical properties of the newly developed product.^15–17^ However, since the grinding process also reduces the length of the reinforcing fibers, the resulting short fibers offer lower reinforcement efficiency compared to continuous fibers, leading to a loss in mechanical strength. Despite this limitation, recycled composites can still be widely applied in products where high performance is not a critical requirement.^18,19^

Furthermore, the increasingly critical requirement for electromagnetic interference shielding effectiveness (EMI-SE) has become an essential design requirement in critical applications, particularly in the aerospace and defense industry. In these sectors, the environment presents high radiation, thermal fluctuations, and electromagnetic waves, and thus, protection against EMI from both internal and external sources is essential, whereas an electronic failure caused by EMI can have catastrophic consequences on aircraft and military vehicles, for example.^20^ Thermoplastic composites have been gaining prominence, in addition to presenting satisfactory mechanical performance, as well as for EMI shielding. Santos et al.^15^ develop hybrid composites by adding CF/epoxy resin waste and carbon nanotubes (CNTs) in epoxy matrix and achieved the highest electromagnetic shielding efficiency (25 dB) in the X-band, demonstrating potential for functional electromagnetic applications. De Oliveira et al.^17^ develop a recyclable composite from protective film (polyethylene) waste by incorporating CF/PAEK composite particles obtained through grinding in knife mills, followed by an analysis of the particle size distribution. The authors observed that the increase in the addition of CF/PAEK composite particles contributed to the increase in the attenuation of the electromagnetic wave.

Although previous studies, including our own (e.g., Montagna et al.;^21^ Oliveira et al.^17^), have investigated the recycling of CFRT waste into thermoplastic matrices, the present work advances the state-of-the-art by offering a comprehensive assessment of the multifunctional performance (rheological, mechanical, electrical, and electromagnetic) of PA6-based composites reinforced with recycled CFRT. Unlike earlier studies that focused primarily on structural or thermal behavior, this study highlights the potential of these recycled composites for advanced functional applications. Moreover, it demonstrates the feasibility of a scalable and industry-relevant processing route, reinforcing the role of mechanical recycling in addressing environmental challenges associated with high-performance composite waste.

## 2 Experimental Section

### 2.1 Materials

Polyamide 6 (PA6), commercial grade Aegis H8202NLB, was supplied by AdvanSix (USA). The material has a density of 1.13 g/cm^3^ and a melt flow index (MFI) of 9.8 g/10 min (235 °C, 2.16 kg). The carbon fiber-reinforced thermoplastic (CFRT) composite waste used in this study was supplied by Toray (UK) under the commercial designation Toray Cetex TC910. This material consists of a five-ply CF/PA6 laminate, comprising 2 × 2 twill woven carbon fiber fabric (415 gsm) with a PA6 content of 39 wt %.

### 2.2 Mechanical Recycling: Grinding Process

The CFRT residues (CF/PA6) generated from the manufacturing processes of automotive components were collected and manually cut into small pieces (approximately 20 mm × 20 mm) using an angle grinder (4 1/2″ G720, 820 W, Black & Decker) equipped with aluminum oxide cutting discs. The material was subsequently ground using a steel knife mill (RONE, model S200) to obtain a homogeneous particle size suitable for further processing.

The particle size of the ground CFRT was analyzed using digital optical microscopy (OM) (Instrutherm, model MP-150). The images were captured and processed using the open-source software ImageJ, through which the lengths of the waste fragments were measured to determine the particle size distribution.

Figure 1 shows the process steps from receipt to grinding of CFRT waste. Figure 1A presents the as-received CFRT laminates; Figure 1B displays the CFRT after the grinding process using a knife mill; and Figure 1C shows the optical microscopy image of the ground CFRT. The average fragment size after the grinding process was 3.3 ± 1.9 mm (Figure 1D), which is suitable for the extrusion process.^14^

**Figure 1:**
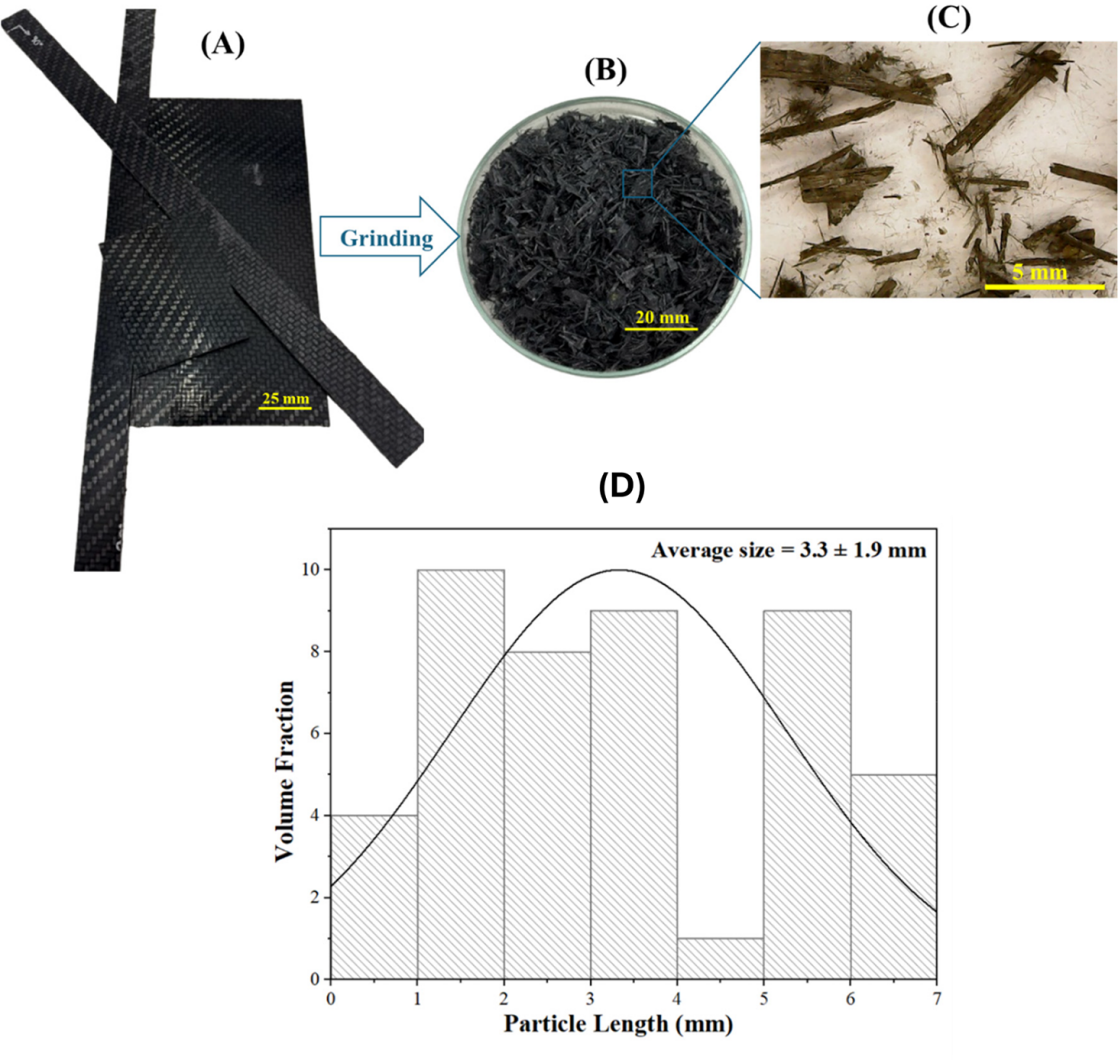
(A) Carbon fiber-reinforced thermoplastic (CFRT) composite waste, (B) CFRT after the grinding process using a knife mill, (C) optical microscopy image of the ground CFRT, and (D) ground CFRT size distribution analysis.

### 2.3 Processing of PA6/Ground CFRT Composites

The PA6 and the ground CFRT were initially dried in a vacuum oven (Sheldon Manufacturing, SVAC1E) for 24 h at 80 °C. Thus, PA6 composites with different contents of ground CFRT (0, 10, and 20 wt %) were prepared by the melt mixing method in a corotational twin-screw extruder (AX Plásticos, model AX16:40DR), with an L/D ratio of 40 and 16 mm of diameter screws. The temperature profile was 205/210/215/215/220 °C, and the feeding and screw rotations were 20 and 80 rpm, respectively. Table 1 presents the terminology and weight percentages of the compositions. The ground CFRT contents used in this study (10 and 20 wt %) were selected based on prior literature^21^ considering that higher filler loadings can lead to excessive material stiffness and increased wear on the extruder components due to the elevated friction between the fiber-rich material and the equipment.

**Table 1:** Samples’ Nomenclature and Compositions

Samples	PA6 (wt %)	Ground CFRT (wt %)	Composite weight ratio (PA6/CF)^a^
PA6	100	-	-
PA6/CFRT (90/10)	90	10	(93.9/6.1)
PA6/CFRT (80/20)	80	20	(87.8/12.2)

a Composite weight ratio considering the composition of the CF/PA6 composite (61/39 wt %).

PA6 and the composite samples were injection-molded using a 270 V ARBURG machine with a temperature profile of 210/220/230/230/240 °C. The process parameters included an injection flow rate of 20 cm^3^·s^–1^, a mold temperature of 80 °C, a back pressure of 30 bar, and a cooling time of 30 s. Specimens for Izod impact strength and tensile (Type I) tests were prepared according to ASTM D256 ^22^ and ASTM D638,^23^ respectively.

### 2.4 Characterization of PA6/CFRT Composites

#### 2.4.1 Field-Emission Gun Scanning Electron Microscopy (FEG-SEM)

The morphologies of the PA6/CFRT composites were analyzed using a TESCAN MIRA3 field-emission gun scanning electron microscope operated at an accelerating voltage of 5 kV. The samples were fractured perpendicularly to the injection molding flow direction using liquid nitrogen, mounted on aluminum stubs with conductive carbon tape, and sputter-coated with a thin layer of gold before imaging.

#### 2.4.2 Rheology

The complex viscosity (η*), shear storage modulus (*G*′), and shear loss modulus (*G*″) were measured under small-amplitude oscillatory shear (SAOS) using a stress-controlled rotational rheometer (ARG2, TA Instruments). The tests were conducted under an inert nitrogen atmosphere using parallel-plate geometry (*D* = 25 mm and gap = 1 mm) at 230 °C, within an oscillatory frequency range of 0.01–500 rad·s^–1^. Before the frequency sweep tests, strain sweep tests were carried out to determine the linear viscoelastic region (LVR), establishing a strain amplitude of 0.5% for all samples.

Measurements of the steady-state shear viscosity (η) were performed using the same rheometer and similar test conditions, within a shear rate range of 0.01–10 s^–1^.

#### 2.4.3 Differential Scanning Calorimetry (DSC)

The thermal properties of the PA6 and PA6/CFRT composites were analyzed by DSC using a TA Instruments Q2000 system, with nitrogen as a purge gas at a constant flow rate of 50 mL·min^–1^. Samples weighing approximately 10 mg were conditioned in sealed aluminum crucibles and were initially heated from 0 to 260 °C at a rate of 10 °C·min^–1^, followed by an isothermal hold at 260 °C for 2 min to eliminate thermal history. Subsequently, the samples were cooled from 260 to 0 °C at 10 °C·min^–1^ and then subjected to a second heating cycle from 0 to 260 °C at the same rate. The degree of crystallinity (*X*_c_) was calculated using eq 1:

1 $$X_{c}\left(\%\right)=\frac{\Delta H_{m}}{W\cdot \Delta H_{m}^{o}}\times 100$$

where Δ*H*_m_ is the melting enthalpy, *W* is the weight fraction of PA6 in the composite, and $\Delta H_{m}^{o}$ is the theoretical enthalpy for 100% crystalline PA6 (190.8 J·g^–1^).^24^

#### 2.4.4 Mechanical Characterization: Tensile Test and Izod Impact Strength Test

Before mechanical testing, all specimens were dried in a vacuum oven at 80 °C for 4 h and subsequently stored in a desiccator to prevent moisture interference.

Tensile tests were performed in an MTS universal testing machine (model Criterion 45) using a crosshead speed of 50 mm/min and a load cell of 50 kN. Five injection-molded specimens, Type I (ASTM D 638 ^23^) were analyzed for each composition.

Izod impact strength tests were performed according to ASTM D256 ^22^ in a CEAST/Instron Izod impactor test machine (model 9050) coupled with a hammer of 5.5 J. The notch in the specimens was done previously with a manual notching machine (CEAST/Instron, model 9050).

Statistical analysis of mechanical properties involved two distinct approaches. First, a one-way analysis of variance (ANOVA) was conducted, followed by post hoc Tukey HSD tests for pairwise comparisons at a significance level of 5% (*p* = 0.05).

#### 2.4.5 Impedance Spectroscopy (IS)

A Solartron SI 1260 impedance analyzer was applied to determine the volumetric electrical resistivity on alternating current (AC) from 3 to 10^6^ Hz by impedance spectroscopy analyses. The impedance measurements were made at a voltage amplitude of 0.5 V, with injection-molded samples measuring in the injection mold direction and at room temperature. The electric contacts consisted of gold electrodes sputtered on both parallel faces of the samples. From the real (*Z*′) and imaginary (*Z*″) impedance measured, the total electrical conductivity (σ) was calculated by eq 2:

2 $$\sigma =\frac{d}{A\left|Z^{*}\right|}$$

where Z* is the complex impedance module (Ohms), *A* is the area of the electrodes, and *d* is the thickness of the sample (3.5 mm).

#### 2.4.6 Electromagnetic Response in a Rectangular Waveguide

The electromagnetic interference shielding effectiveness (EMI-SE) behavior was evaluated in the X-band frequency range (8.2–12.4 GHz) in a vector network analyzer (VNA, Agilent Technologies, model PNA-L N5235A) coupled with a waveguide (WR-90). The complex relative permittivity (ε_r_) and relative permeability (μ_r_) were calculated using the Nicolson–Ross–Weir (NRW) mathematical model. The sample thickness was 3.5 mm. The coefficients of reflection (*R*), absorption (*A*), and transmission (*T*) of the electromagnetic wave, as well as the total shielding effectiveness (SE_T_), reflection shielding effectiveness (SE_R_), and absorption shielding effectiveness (SE_A_), were calculated according to eqs 3–8 from the complex scattering parameters *S*_11_ (reflected power) and *S*_21_ (transmitted power). The multiple reflection shielding effectiveness (SE_M_) mechanism was neglected.

3 $$T={|S_{21}|}^{2}$$

4 $$R={|S_{11}|}^{2}$$

5 T=1−R−A

6 $${\mathrm{SE}}_{T}=10\log_{10}\left(\frac{1}{T}\right)$$

7 $$SE_{R}=10\log_{10}\left(\frac{1}{1-R}\right)$$

8 $$SE_{A}=10\log_{10}\left(\frac{1-R}{T}\right)$$

## 3 Results and Discussion

### 3.1 Characterization of the PA6/CFRT Composites

The incorporation of ground CFRT into the PA6 matrix was evident from the presence of short carbon fibers within the structure (Figure 2A–D). These fibers were further identified by the occurrence of fiber pull-outs and indentations resulting from the cryofracture process. Notably, the fibers did not exhibit a preferential orientation, as indicated by their random distribution and varying angles throughout the matrix. At higher magnifications, the matrix revealed a continuous phase, with no distinct separation between the original PA6 that surrounded the fibers in the ground CFRT and the neat PA6 used as the matrix in the composite. This observation is expected since both phases consisted of the same PA6 grade and remained miscible after extrusion.

**Figure 2:**
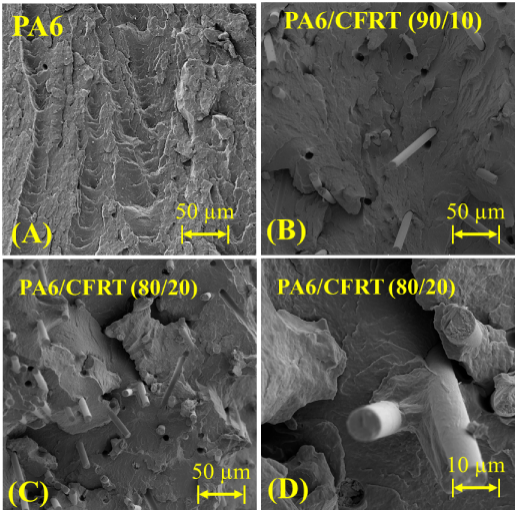
FEG-SEM microscopy of (A) the PA6 matrix, (B) PA6/CFRT (90/10) composites, and (C and D) the PA6/CFRT (80/20) composites.

The continuity of the matrix observed in SEM may be explained because during the extrusion process, the PA6 present on the surface of the CF is likely mixed with the neat PA6, leading to the formation of strong intermolecular interactions, such as hydrogen bonding and molecular entanglements inherent to PA6 chains characteristic of this polymer.^25,26^ These interactions are expected to contribute to the enhanced mechanical properties of the PA6/CFRT composites, as the improved adhesion between PA6 and CF promotes more efficient stress transfer across the interface. This effect aligns with the findings of Botelho et al. (2003), who highlighted the critical role of interfacial shear strength and fiber–matrix adhesion in determining the macroscopic mechanical behavior of carbon fiber-reinforced composites.^27^

The rheological behavior of the PA6/CFRT composites can also be correlated with their morphological characteristics (Figure 3B–D). The complex viscoelastic response of the composites is attributed to the interaction between the CF and the matrix. As expected, the addition of 10 and 20 wt % ground CFRT resulted in a proportional increase in complex viscosity (η*) and both components of the complex shear modulus (*G*′ and *G*″). These increases can be directly associated with the restricted movement of the PA6 chains caused by the presence of solid CF in the matrix, particularly at the surface of the fillers.^28^ The effect observed for the addition of 20 wt % ground CFRT (12.2 wt % effective CF content) was relatively modest, likely due to the micrometric size of the CF, which has a lower aspect ratio compared to the nanomaterials, such as multiwalled carbon nanotubes (MWCNTs), that typically induce more pronounced rheological changes even at lower contents.^29,30^

**Figure 3:**
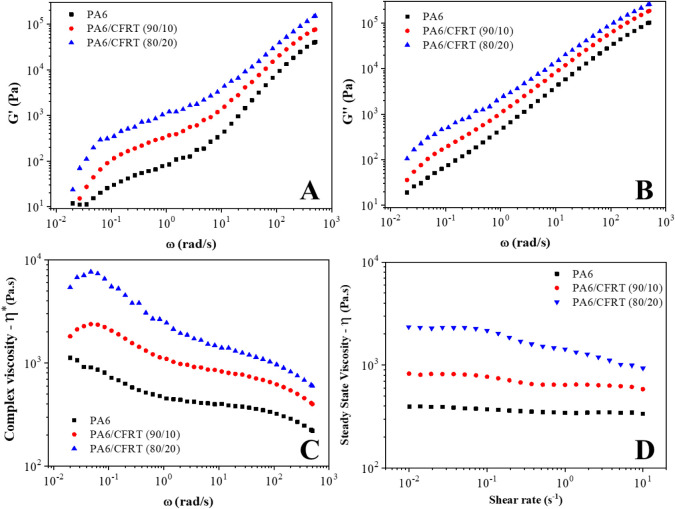
Rheological characterization of the PA6 and the composites: complex shear modulus (G*). (A) Storage modulus (*G*′), (B) loss modulus (*G*″), (C) complex viscosity (η*), and (D) steady-state viscosity (η).

In terms of steady-state viscosity, the behavior observed in Figure 3D is advantageous from a processing perspective, as the increase in the viscosity was minimal. This observation is consistent with the findings of Liu et al. (2024), who investigated the viscosity behavior of short carbon fiber-reinforced PA6 using capillary rheometry. They reported that the self-lubricating effect of short CF limited the increase in viscosity, with only a noticeable change observed at low filler contents. In contrast, composites containing 20–40 wt % CF exhibited similar viscosity behavior.^31^

In the steady state, PA6 exhibited a superior Newtonian plateau, which was only slightly elevated upon the addition of ground CFRT. The Cox–Merz rule appears to be less applicable in this system, as indicated by the discrepancies between the complex viscosity and steady-state viscosity results.^28^ Although PA6 is typically considered a linear homopolymer, the presence of intermolecular interactions, such as hydrogen bonding, may have contributed to deviations from the expected oscillatory behavior, affecting both the complex viscosity and the variation in *G*′ at lower frequencies for PA6. An increase in ground CFRT content from 10 to 20 wt % resulted in higher values of storage (*G*′) and loss (*G*″) moduli and a slight reduction in the frequency dependence of *G*′, suggesting a tendency toward more solid-like behavior associated with the development of a fiber-reinforced structure in the PA6 matrix.^28^

Figure 4 shows the DSC curves, and the corresponding thermal data are summarized in Table 2. A comparison of the first and second heating cycles of the neat PA6 reveals that the glass transition temperature (*T*_g_) increased from 45 to 53 °C. This change can primarily be attributed to the effect of moisture on PA6. Water molecules, which may be adsorbed from the atmosphere, are known to have a plasticizing effect on PA6. They interfere with the hydrogen bonding between the chains, reducing intermolecular interactions and increasing chain mobility. During the first heating cycle, most of these water molecules are likely eliminated at higher temperatures, which reduces their plasticizing effect. As a result, the second heating cycle exhibits a higher *T*_g_ value, which is closer to the supplier’s specified value of 60 °C.

**Figure 4:**
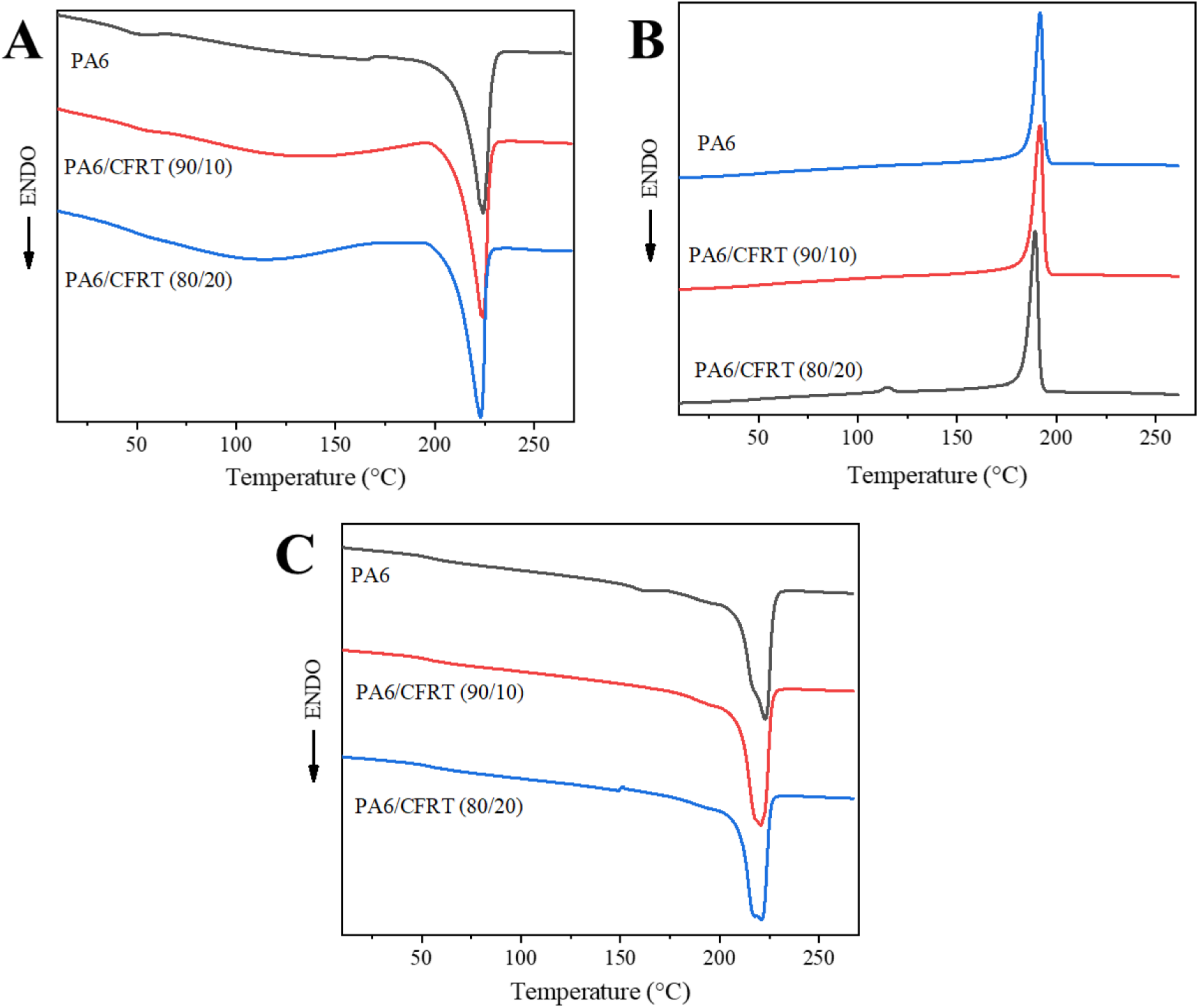
DSC curves of PA6/CFRT composites: (A) first heating, (B) cooling, and (C) second heating.

**Table 2:** Thermal Characteristics of PA6 and PA6/CFRT Composites

	Samples	PA6	PA6/CFRT (90/10)	PA6/CFRT (80/20)
**1° Heating**	*T*_g_ (°C)	45	47	46
	*T*_m_ (°C)	224	224	223
	Δ*H*_m_ (J/g)	57.4	56.2	54.6
	Δ*H*_m/PA6_ (J/g)	57.4	59.9	62.2
	*X*_c_ (%)	30.1	31.4	32.6
**Cooling**	*T*_c_ (°C)	189	192	192
**2° Heating**	^2^ *T*_g_ (°C)	53	53	54
	^2^ *T*_m_ (°C)	223	221	221
	^2^Δ*H*_m_ (J/g)	51.1	59.6	55.5
	^2^Δ*H*_m/PA6_ (J/g)	51.1	63.5	63.2
	^2^ *X*_c_ (%)	26.8	33.3	33.1

The addition of ground CFRT to the composites likely causes the CF surfaces to act as heterogeneous nucleation sites. This behavior promotes crystallization phenomena that initiate at higher temperatures and facilitates the formation and growth of more crystalline regions. These effects are reflected in the results, with the crystallization temperature (*T*_c_) increasing by approximately 3 °C for the CFRT compositions and the degree of crystallinity (*X*_c_) being significantly higher compared to neat PA6. Additionally, this nucleating effect may also explain the slightly lower melting temperature (*T*_m_) observed during the second heating cycle. The crystalline lamellae formed are likely smaller due to the heterogeneous nucleation, resulting in melting at a slightly lower temperature (221 °C compared to 223 °C for PA6). These observations are consistent with the findings of An et al.,^32^ who reported increased crystallization temperatures in long CF/PA6 composites due to the heterogeneous nucleating effect of CF, with negligible changes in melting behavior.

While these changes in *T*_c_ and *T*_m_, in the order of a few degree Celsius, are interesting from a scientific standpoint, they are generally not significant from an application perspective. These thermal property results corroborate findings by Karsli and Aytac,^33^ who also reported increased crystallinity in CF/PA6 composites, likely driven by heterogeneous nucleation, without significant changes in melting or glass transition temperatures.

As shown in Figure 5A, the incorporation of ground CFRT into the PA6 matrix significantly improved its mechanical properties, particularly the elastic modulus (*E*) and ultimate tensile strength (UTS), transforming PA6 from a ductile material into one that is stiffer and stronger. This shift, accompanied by reduced strain at break, indicates strong interfacial bonding between the CF in the CFRT and the PA6 matrix, as also evidenced by the PA6/CFRT (80/20) morphology (Figure 2). The interdiffusion of polymer chains and the strong intermolecular interactions between the PA6 surrounding the CF and the added PA6 matrix contribute to excellent load transfer during testing.

**Figure 5:**
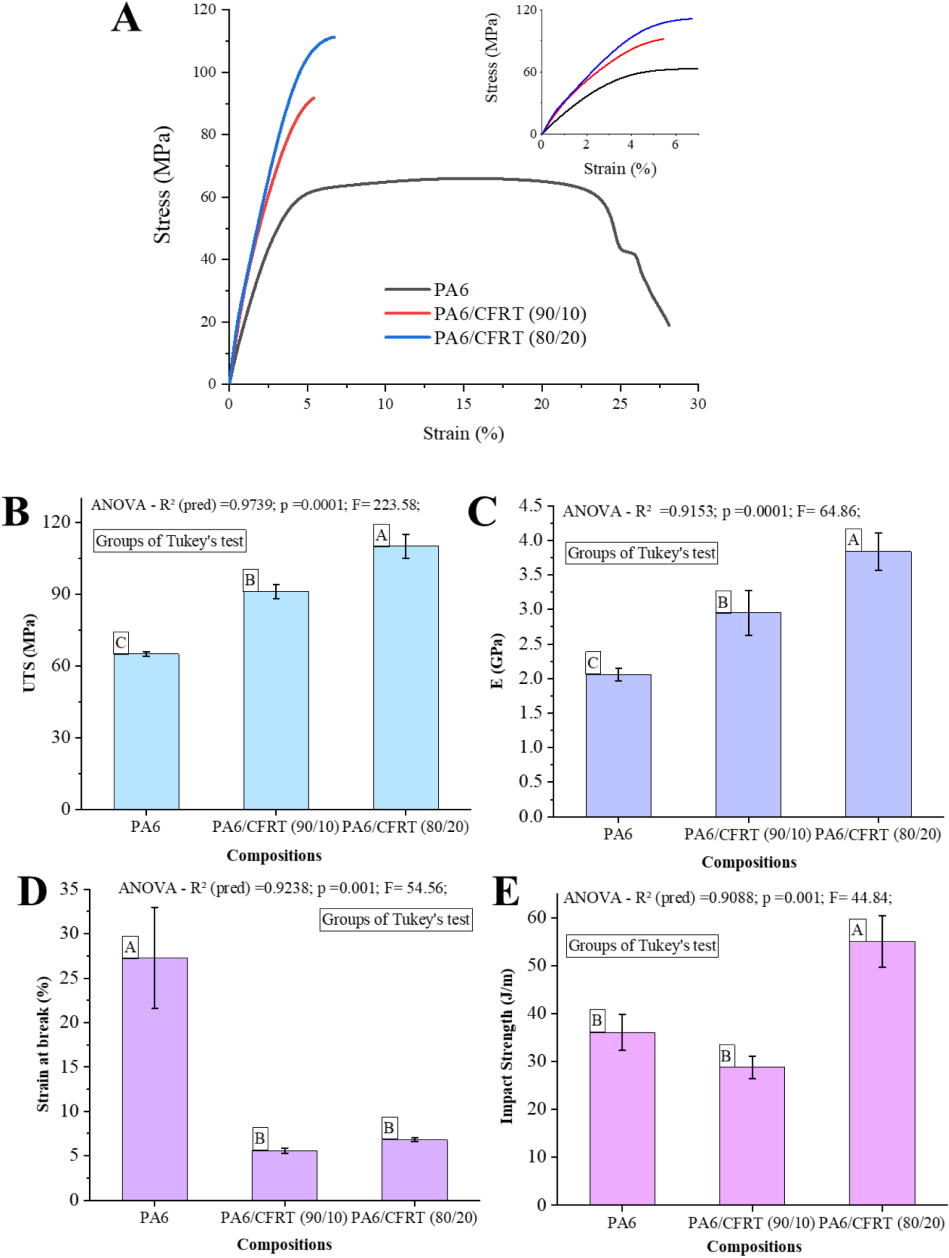
(A) Stress–strain curves, mechanical test results, and statistical treatment, (B) ultimate tensile strength (UTS), (C) elastic modulus (*E*), (D) strain at the break, and (E) Izod impact strength.

The increase in CF content, proportional to the ground CFRT addition, led to a corresponding rise in both UTS and *E*, as observed in Figure 5B,C. The addition of 20 wt % ground CFRT resulted in a 69% increase in UTS and an 80% increase in *E*, reaching average values of 110 MPa for UTS and 3.8 GPa for *E*. These values are interesting for an engineering thermoplastic being characteristic of the short CF/PA6 composites^34–36^ and highlighting the potential of this recycling strategy. Statistical analysis using ANOVA followed by Tukey’s test confirmed that the different compositions exhibited statistically significant differences, with both UTS and *E* improving as the ground CFRT content increased.

It is worth noting that, although a direct reference using the same recycling process, CF content, and CF length distribution is not available, the obtained values were comparable to those reported for nonrecycled short CF/PA6 composites at higher CF contents. For instance, Li et al. (2022) reported a UTS of ∼167 MPa and a modulus of 6.3 GPa for 3D-printed short CF/PA6 with 25 wt % CF.^35^ Similarly, Sun et al.^34^ reported a UTS of 163 MPa and a modulus of ∼4 GPa at the same fiber content. More recently, Dong et al.^36^ described UTS values ranging from 160 to 170 MPa for composites with an approximate fiber content of 23–25 wt %.

Conversely, for *E* and UTS, the strain at break significantly decreased, which can be attributed to the presence of interfaces with the CF. The presence of a strong interface makes the composite material more rigid, thus with less deformation when subjected to mechanical stress. This explains the reduced maximum strain of approximately 5% in the composites, which exhibit a stiffer and less ductile behavior. In this context, the fracture mechanism of PA6 likely shifted from shear yielding, responsible for dissipating significant energy and promoting high toughness in neat semicrystalline engineering polymers such as PA6, to a predominantly crazing-driven mechanism in the composites, contributing to a more brittle response. These trends in tensile properties are consistent with previous studies on short CF-reinforced PA6, which have reported similar improvements in tensile modulus and strength, accompanied by reduced strain at break due to restricted chain mobility and stress concentration around fiber–matrix interfaces.^33^

Figure 5D shows the Izod impact strength results of the compositions. Neat PA6 is known to exhibit low impact strength in the presence of a notch, around 36 J/m. However, the impact strength improved with higher ground CFRT content (20 wt %), reaching 55 J/m, representing a 53% increase. This enhancement can be attributed to the toughening effect of the CF within the PA6 matrix, which, during impact, may contribute to energy dissipation through mechanisms such as crack deflection, crack branching, and fiber pull-out. These mechanisms lead to greater energy absorption and, consequently, increase impact resistance. It is important to note, however, that this improvement is content-dependent, as no significant improvement was observed at lower ground CFRT content. This behavior aligns with the findings of Molnár et al.,^37^ who reported that low CF contents in PA6 composites tend to promote crack propagation. In contrast, higher CF contents enhance impact resistance by activating energy dissipation mechanisms like fiber pull-out and crack deflection.

Several methods have been proposed to estimate the effect of fibers on mechanical properties, such as the elastic modulus and ultimate tensile strength. These include recently developed complex microstructure-based models, such as the one proposed by Gao et al.,^38^ as well as some simpler, traditional theoretical models.^39^ Figure 6 presents a comparison between experimental data and predictions from some of these models. The theoretical values were calculated using the average experimental data for neat PA6 (*E*_PA6_ = 2.1 GPa and UTS_PA6_ = 65 MPa) and the datasheet values for the CFRT along the fiber axis (0°),^40^ which are *E*_CF_ = 99.9 GPa and UTS_CF_ = 2048 MPa. The weight fractions (ϕ) used are described in Table 1, where ϕ_PA6_ = 1 – ϕ_CF_.

**Figure 6:**
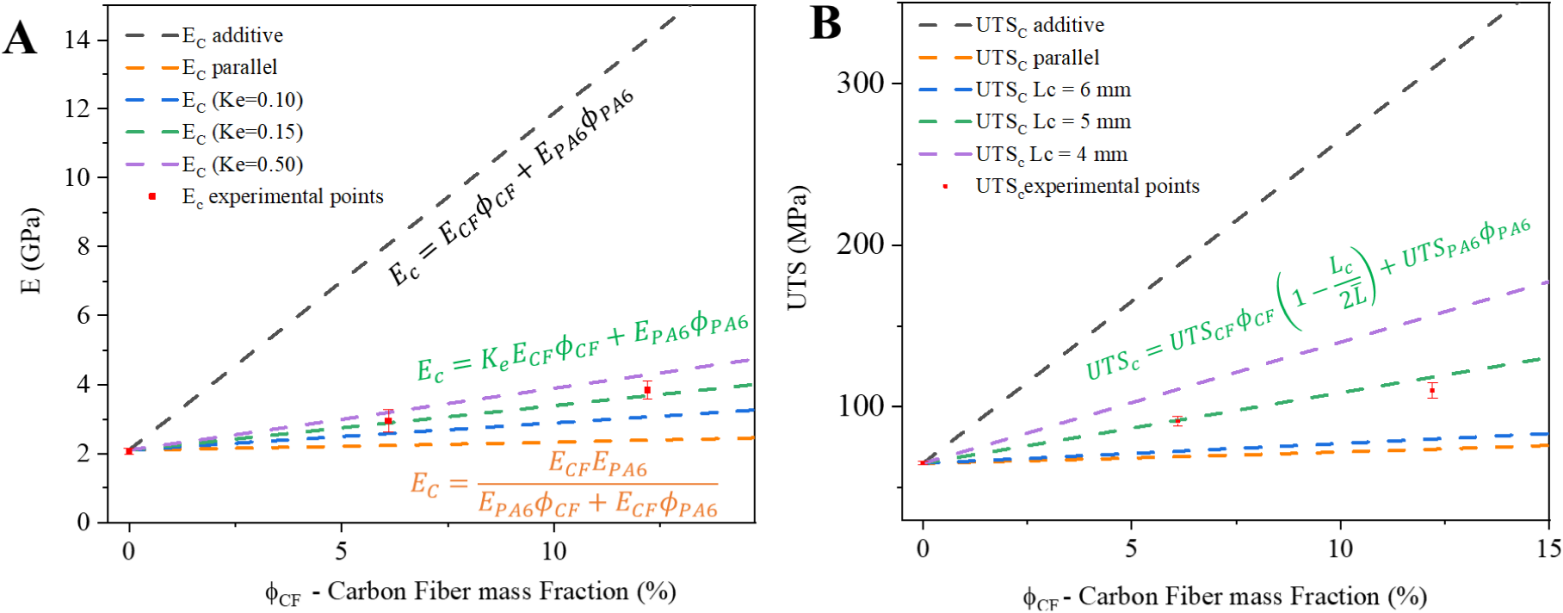
Empirical models for (A) elastic modulus (*E*) and (B) ultimate tensile strength (UTS) of the PA6/CFRT composites.

The two most common and straightforward models are parallel to the fiber-axis-direction model, which, for continuous fiber-reinforced polymer composites, applies to the direction aligned with the fiber axis (eq 9, black dashed lines in Figure 6), and perpendicular to the fiber-axis-direction model (eq 10, orange dashed lines in Figure 6), which is used in the same context to estimate the elastic modulus (*E*) in the direction parallel to the fiber axis.^39^

9 $$E_{c}=E_{CF}\phi_{CF}+E_{PA6}\phi_{PA6}$$

10 $$E_{C}=\frac{E_{CF}E_{PA6}}{E_{PA6}\phi_{CF}+E_{CF}\phi_{PA6}}$$

For composites with nonoriented discontinuous fibers, these two models represent the upper and lower bound cases, respectively. Most of these composites exhibit mechanical behavior closer to the lower bound. In a more realistic scenario, the reinforcing effect of the CF addition is influenced by factors such as fiber misorientation and finite fiber length. This behavior can be accounted for by introducing a fiber efficiency factor (*K*_e_) to estimate the composite modulus (*E*_C_), as shown in eq 11.

11 $$E_{C}=K_{e}E_{CF}\phi_{CF}+E_{PA6}\phi_{PA6}$$

As illustrated in Figure 6A, the experimental values correspond to a *K*_e_ value of approximately 0.15, which is consistent with values reported in the literature, typically around 0.1 for randomly oriented discontinuous fibers.^39^ For UTS, the reinforcing effect can also be estimated and is influenced by the average fiber length (*L̅* = 3.3 mm; Figure 1), which must be compared to the critical fiber length (*L*_c_) required to effectively transfer stress and promote the reinforcing effect, as described in eq 12.

12 $$UTS_{c}=UTS_{CF}\phi_{CF}\left(1-\frac{L_{c}}{2\mathrm{L̅}}\right)+UTS_{PA6}\phi_{PA6}$$

Considering the experimental values obtained, the behavior indicates a critical fiber length (*L*_c_) of approximately 5 mm, which is required to achieve effective reinforcement and approximately fit the data points.

CF are composed of sp^2^-hybridized carbon atoms arranged in a hexagonal lattice structure aligned along the fiber axis.^41^ This highly ordered structure not only imparts excellent mechanical strength but also increases electrical conductivity.^15,17^ When dispersed in an insulating polymer matrix such as PA6, CF can significantly enhance the electrical properties of the resulting composite. This effect is confirmed by the results in Figure 7, which show that the AC volumetric electrical conductivity of PA6 increases proportionally with the added ground CFRT content, shifting the entire conductivity curve upward.

**Figure 7:**
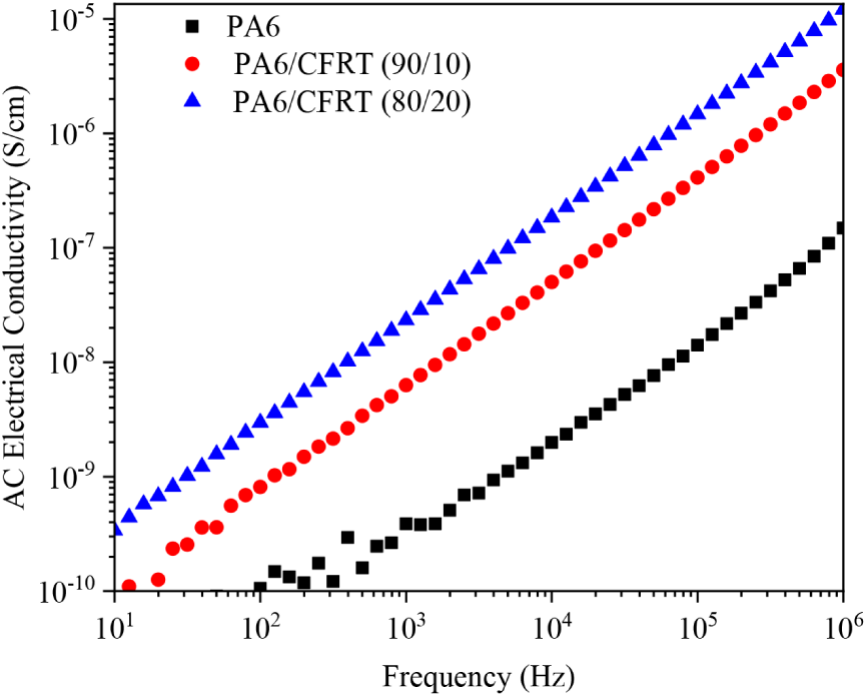
AC volumetric electrical conductivity for the PA6 and the PA6/CFRT composites obtained by impedance spectroscopy.

However, the composites still behave as insulating materials, with conductivity values increasing linearly with frequency. This behavior may be attributed to the relatively low CF contents (∼6.1 and 12.2 wt %) and the micrometric size of the filler. As reported in previous studies,^15,17,21^ the electrical performance of CF/polymer composites is highly dependent on their morphology, including fiber dispersion, orientation, and the development of percolating conductive networks. From this, and as suggested in previous studies, achieving conductive behavior and establishing electrical percolation paths would require a higher CF content^21^ or the incorporation of small amounts of other conductive fillers, such as carbon nanotubes.^15^

Polymer composites with conductive carbon-based fillers are of particular interest for electromagnetic interference shielding efficiency (EMI-SE) applications in the electronics and telecommunication industries, as well.^41^ The electromagnetic response of 3.5 mm thick samples (Figure 8) demonstrated promising shielding performance. The PA6/CFRT composites exhibited a shielding absorption (SE_A_) component, primarily attributed to interfacial polarization and ohmic loss mechanisms resulting from the semiconducting nature and spatial arrangement of the CF.^41^ This SE_A_ component increased proportionally with the CF content in the composites. The reflection-related shielding component (SE_R_) exhibited frequency-dependent behavior, being lower than that of neat PA6 over certain frequency ranges of 9.8–12.1 GHz for PA6/CFRT (90/10) and 8.2–9.1 GHz for PA6/CFRT (80/20). This reduction is likely associated with the heterogeneous morphology of the composites, which affects impedance matching.

**Figure 8:**
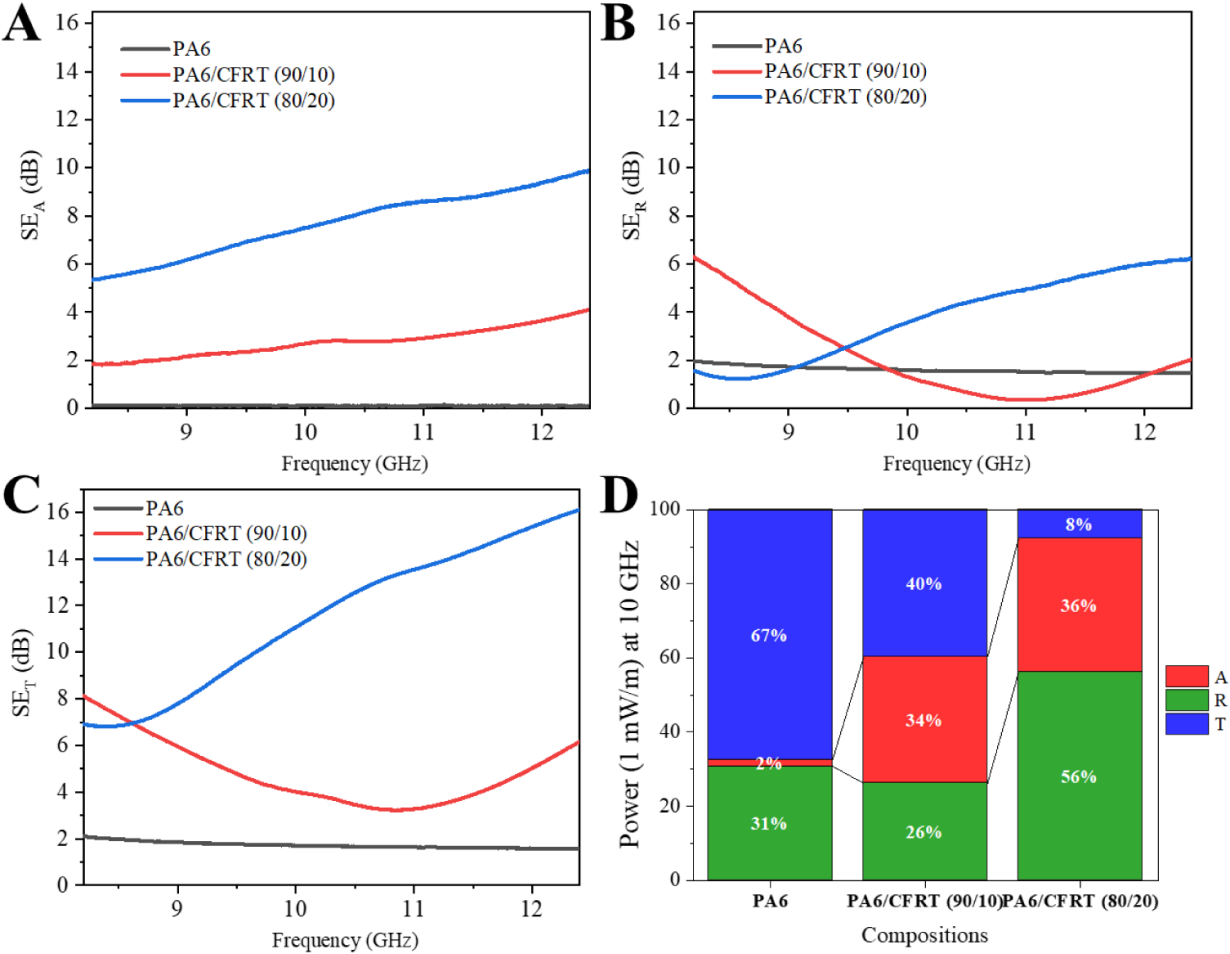
Electromagnetic interference shielding efficiency (EMI-SE) response for 3.5 mm thick samples, in terms of (A) absorption efficiency (SE_A_), (B) reflection efficiency (SE_R_), (C) total shielding efficiency (SE_T_), and (D) power coefficients (*A*, *R*, and *T*) distribution at 10 GHz.

Overall, the total shielding effectiveness (SE_T_) was predominantly affected by changes in the SE_A_ component and showed a strong correlation with CF content. At higher frequencies in the X-band, the SE_T_ reached approximately 16 dB, corresponding to 97% attenuation of electromagnetic wave energy near 12.4 GHz. Although these results are interesting and modest, they still fall below the benchmark typically required for shielding applications, which is around 10 dB/mm (or ∼20 dB for a 2 mm sample).^30^ To achieve such levels of attenuation, similar to the requirements for electrical conductivity, it would be necessary to either increase the CF content^21^ or incorporate hybrid fillers.^15^

The same trends were observed in the power coefficient distribution at 10 GHz, where the transmission coefficient (*T*) progressively decreased with increasing ground CFRT content. Similarly, the absorption coefficient (*A*) increased from approximately 2–36% with the addition of 20 wt % ground CFRT. These results agree with Santos et al.’s^15^ data obtained for recycled CF reinforcing epoxy resin fragments obtained by mechanical recycling, dispersed in neat epoxy.

The complex electromagnetic properties, including electric permittivity (ε) and magnetic permeability (μ), were also determined using the Nicolson–Ross–Weir (NRW) method (Figure 9). This method assumes that the samples are homogeneous and exhibit low dielectric and magnetic losses, conditions that were not fully met by the samples evaluated in this study. These limitations likely explain the occurrence of nonphysically justified negative values for the imaginary part of the permittivity (ε″) and the deviations observed in the real (μ′) and imaginary (μ″) components of permeability, which were theoretically expected to be close to 1 and 0, respectively, for nonmagnetic materials.

**Figure 9:**
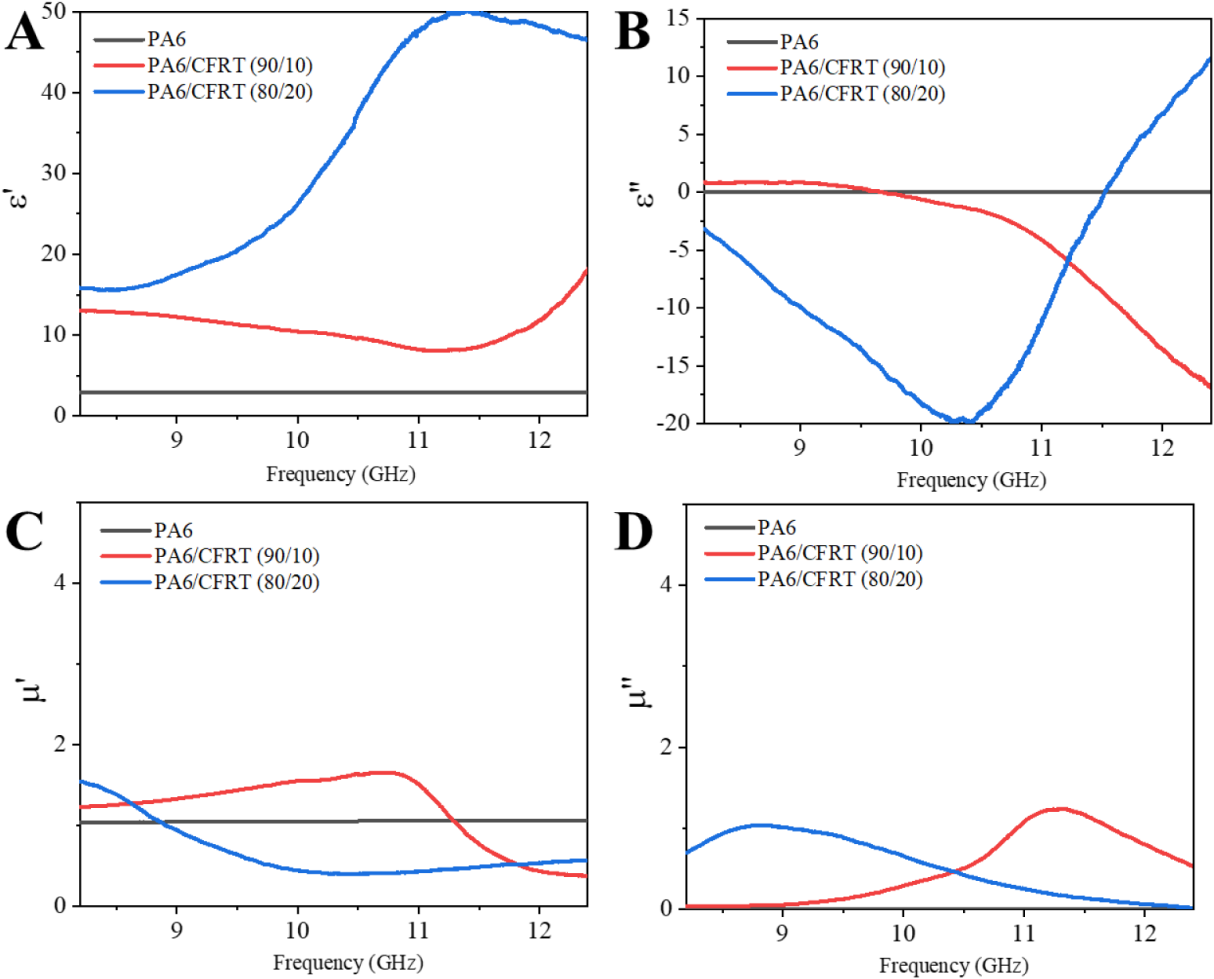
Complex electromagnetic properties according to the NRW model: (A) real permittivity (ε′), (B) imaginary permittivity (ε″), (C) real permeability (μ′), and (D) imaginary permeability (μ″).

Although this study primarily targets the technical feasibility of mechanically recycling CFRT waste into PA6 composites, recent life-cycle assessment (LCA) data underscore the environmental relevance of this approach. The production of polyacrylonitrile (PAN)-based carbon fibers exhibits a carbon footprint ranging from approximately 13–34 kg CO_2_-eq per kilogram of fiber, depending on production scenarios and energy sources. Furthermore, mechanical recycling routes for CFRP have been shown to achieve reductions in global warming potential (GWP) of up to about 19–27 kg CO_2_-eq per kg of composite, compared to landfill or incineration.^42^ By reintegrating roughly 200 g of CFRT waste per kilogram of PA6 composite, our method contributes not only to diverting material from disposal but also to reducing demand for virgin carbon fibers, reinforcing its potential to support more sustainable, circular high-performance materials.

## 4 Conclusions

This study demonstrated the technical viability and multifunctionality of PA6 composites reinforced with recycled CFRT waste. From a processing perspective, rheological analyses indicated that the incorporation of ground CFRT had minimal impact on melt flow behavior, preserving good processability. Thermal analysis revealed a heterogeneous nucleating effect of carbon fibers on the PA6 matrix, promoting increased crystallinity.

Mechanically, the addition of ground CFRT significantly enhanced the elastic modulus and tensile strength of the composites, while also improving impact resistance at higher reinforcement levels (20 wt %). These enhancements were attributed to effective fiber–matrix interfacial interactions and the reinforcing role of short carbon fibers dispersed within the polymer.

In terms of electrical and electromagnetic performance, the composites exhibited improved AC volumetric conductivity and promising EMI shielding effectiveness, particularly in the X-band range. These effects were primarily governed by absorption mechanisms associated with conductive pathways and interfacial polarization.

Altogether, the results confirm that recycled CFRT-reinforced PA6 composites offer a compelling combination of structural performance and EMI shielding, making them suitable for applications in electronics enclosures, automotive parts, and telecommunication components.

This recycling strategy not only enables the valorization of industrial composite waste but also contributes to the development of sustainable, high-performance materials, reinforcing the transition toward a circular and low-carbon economy.
